# Potential Feed Additives as Antibiotic Alternatives in Broiler Production

**DOI:** 10.3389/fvets.2022.916473

**Published:** 2022-06-17

**Authors:** Habtamu Ayalew, Haijun Zhang, Jing Wang, Shugeng Wu, Kai Qiu, Guanghai Qi, Ayalsew Tekeste, Teketay Wassie, Demissie Chanie

**Affiliations:** ^1^Laboratory of Quality and Safety Risk Assessment for Animal Products on Feed Hazards (Beijing) of the Ministry of Agriculture and Rural Affairs Feed Research Institute, Chinese Academy of Agricultural Sciences, Beijing, China; ^2^College of Veterinary Medicine and Animal Sciences, University of Gondar, Gondar, Ethiopia; ^3^Key Laboratory of Agro-Ecological Processes in Subtropical Region, National Engineering Laboratory for Pollution Control and Waste Utilization in Livestock and Poultry Production, Hunan Provincial Engineering Research Center for Healthy Livestock and Poultry Production, Institute of Subtropical Agriculture, Chinese Academy of Sciences, Changsha, China

**Keywords:** antibiotic alternatives, broiler, feed additives, phytogenic, prebiotic, probiotic

## Abstract

This article aimed to describe the current use scenario, alternative feed additives, modes of action and ameliorative effects in broiler production. Alternative feed additives have promising importance in broiler production due to the ban on the use of certain antibiotics. The most used antibiotic alternatives in broiler production are phytogenics, organic acids, prebiotics, probiotics, enzymes, and their derivatives. Antibiotic alternatives have been reported to increase feed intake, stimulate digestion, improve feed efficiency, increase growth performance, and reduce the incidence of diseases by modulating the intestinal microbiota and immune system, inhibiting pathogens, and improving intestinal integrity. Simply, the gut microbiota is the target to raise the health benefits and growth-promoting effects of feed additives on broilers. Therefore, naturally available feed additives are promising antibiotic alternatives for broilers. Then, summarizing the category, mode of action, and ameliorative effects of potential antibiotic alternatives on broiler production may provide more informed decisions for broiler nutritionists, researchers, feed manufacturers, and producers.

## Introduction

The poultry sector is one of the largest food industries in the globe (1). In the near future, by 2050, it would be projected to be 121% of the year 2005 production (2). It has continual growth and industrialization in many parts of the world (3). Particularly, broiler production has shown exponential growth in global meat consumption and business profit, which will be higher in the next century (4–6). This could be because of its comparative advantages including good quality of nutrition, delicious taste, low-fat content, short production period, low production cost, rapid economic progress, and affordable price even for poor levels of society (7, 8). The production has ascended from 9 to 132 million tons in the year range of 1961 to 2019 (3). Seventeen percent of global output is produced in the United States, which is the world's largest poultry meat producer followed by China and Brazil (3).

Per capita, meat consumption has been an increase in the world in which poultry meat accounts 70% of total meat consumed. Over 66 billion broilers are slaughtered in the world each year (9). From these amounts of slaughtered birds, nearly 110 million tons are produced per annum. Per capita broiler meat consumption is higher in developed countries (10). For example, the average broiler meat consumption per capita in the United States, Brazil, and China is 48, 44.2, and 8.3 kg/head/yr, respectively, in 2017 (11). These exponential broiler meat demands are an alarm to boost production.

Antibiotics have been used for many decades in the poultry industry to enhance production, promote growth performance, and protect birds from pathogenic microbes (12–16). For example, supplementation of broilers' diet with antibiotics could increase body weight gain by 5.8 % (17). This improvement was explained by improved appetite and feed conversion efficiency, stimulation of the immune system, and increased vitality and regulation of the intestinal microflora (18).

Antibiotics are also important for fighting infectious pathologies (16, 19, 20) such as necrotic enteritis and coccidiosis (21). Broadly, antibiotics are used in phytosanitary treatments, feed additives, and prophylactic treatments in animals and humans.

Despite its important role, improper uses of antibiotics in animal farming have been reported to increase antimicrobial resistance bacteria as a public health threat (22–24), residues in animal products, and cause environmental pollution (25, 26). Consequently, the use of antibiotics as growth promoters was banned by the European Union in 2005 (27) and China in 2020 (28). To minimize health risks, consumers have great preferences for conventional broiler meat, resulting in shift to antibiotic-free broiler meat production around the globe (13, 14). The ban on antibiotic use, combined with consumers' preferences, provoked scholars to look for antibiotic alternatives (29). This is important to apply sustainable feeding strategies of potential antibiotic alternatives for increasing antibiotic-free broiler meat production (30, 31). Therefore, this review aimed to explain the current use scenario, mode of action, ameliorative effects, and feeding strategies of different antibiotic alternatives including phytogenic groups (marine algae, herbs, plant extract, and essential oils), prebiotics, probiotics, and enzymes in broiler production.

## Current Scenario of Antibiotic Use

The intensity of using antibiotics could vary among nations (32). China is among the world's leading antibiotic producers and consumer, particularly in livestock products (4, 33). This was supported by Ziping (34), who reported that antibiotic use in China is 5 times higher than the international average. Although antimicrobial use in animal production in China increased until 2014, it has fallen in recent years (34). Antimicrobial consumption is projected to be 67% by 2030 and nearly double in Russia, Brazil, China, India, and South Africa (4).

Although antimicrobial consumption in livestock has received little attention, an expert opinion suggests that global consumption of antimicrobials in animals is twice more than in humans (4, 35). In many countries, most commercial broiler producers have reported antibiotic use, i.e., in Ghana (97%) (16), Nepal (90%) (36), Nigeria (89%) (37), Bangladesh (98%) (38), and the United States (40%) (39). Broiler farm intensification could be a driving force for the use of antibiotics as feed additives in developed countries, whereas increasing demand for poultry meat and eggs for food security could be a factor in the developing world and may lead to the risk of developing antibiotic-resistant microbes (40–43).

Globally, the most commonly applied antibiotics to food animal production include tetracyclines, sulfonamides, and penicillins (44). However, this review finds that there are differences in using antibiotics types in different nations that might be due to antibiotic-producing capability, access, price, and banned antibiotics policy platform. Tetracycline, aminoglycosides, penicillins, and fluoroquinolones in Ghana (45), tetracycline, penicillins, and sulfonamides in South Africa (46), bacitracin, tylosin, tetracycline, salinomycin, virginiamycin, and bambermycin in North America (29), and erythromycin, penicillins, tylosin, tetracycline, and vancomycin in China (34) are commonly used antibiotics.

Although the use of antibiotics has ameliorative effects as mentioned above, it has been banned for a decade in different countries because of potential development of antibiotic-resistant human pathogenic bacteria (15, 47, 48). The European Union (EU) has banned non-therapeutic antibiotics used as growth promoters and feed additives in animal production since 2006 (42). Although the ban was applied before a decade and consumers have preferred organic livestock products, antibiotics are still used in livestock as growth promoters. Therefore, feed additives could be familiar as antibiotic alternatives in the poultry production sector, with a great interest in improving growth performance and feed conversion ratio, maintaining healthy intestinal microbial populations, and improving the overall health of birds (20, 49–52).

## Feed Additives as Antibiotic Alternatives

**Figure 1:**
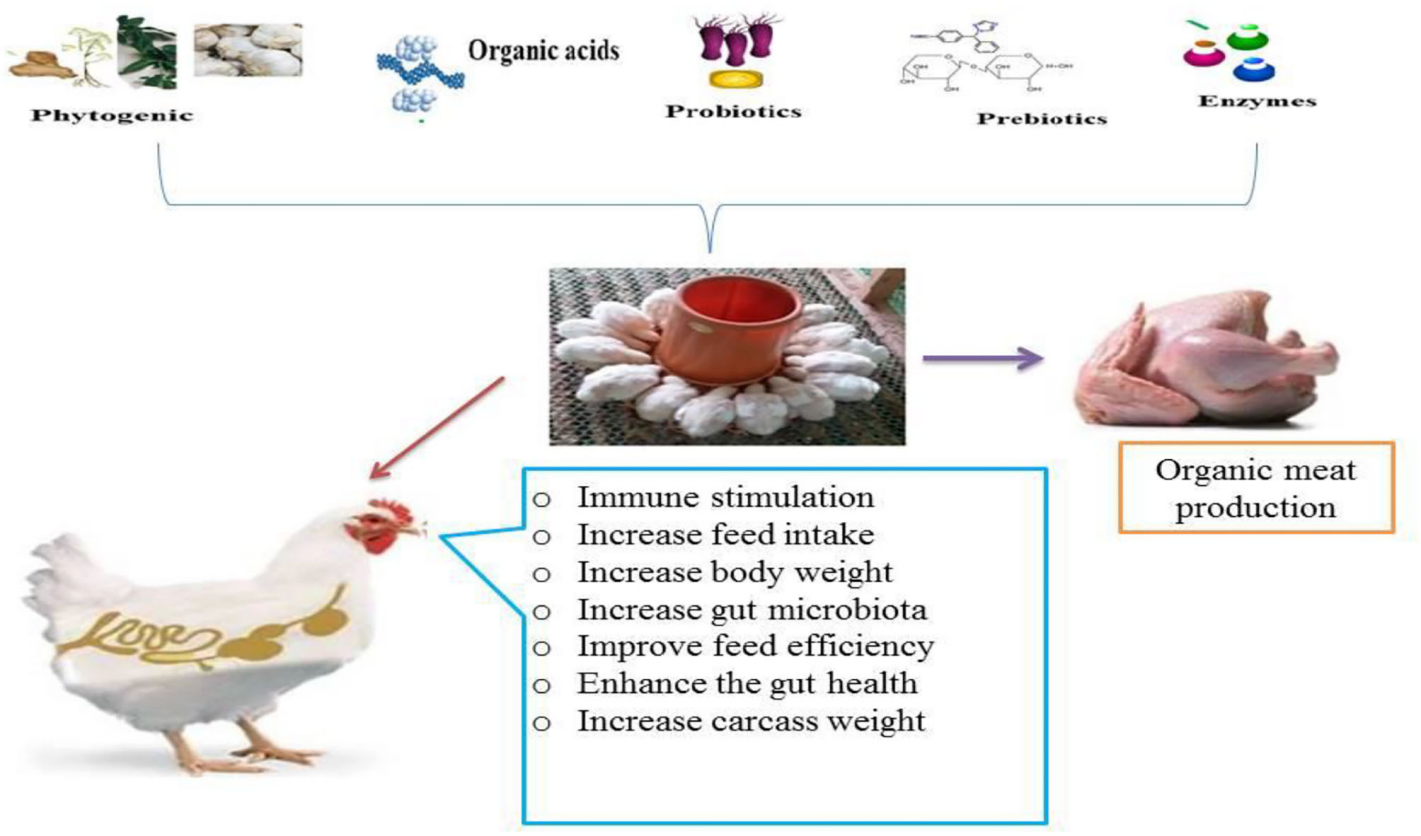
Schematic diagram of an alternative feed additive in broiler diet.

Feed additives are non-nutritive natural products added to basal diet as minor components of the diet to improve feed quality and food from animal origins and improve animal performance and health. They also promote ingestion, absorption, nutrient assimilation, and growth of animals by affecting physiological processes such as immune function and stress resistance (53). It has been reported that feed additives could be used as antibiotic alternatives for broilers to reduce mortality rates and enhance performance without jeopardizing the environment and consumer health (20). The common feed additives tested in poultry are phytogenic feed additive groups including essential oils (20, 51, 52, 54), herbal extracts (55–57), organic acids (58, 59), and others like prebiotics (15, 60), probiotics (15, 61), and enzymes (62–64) (Figure 1).

### Phytogenic Feed Additives

Phytogenic feed additives are plant-origin extracted compounds that include a wide range of substances such as herbs, spices, botanicals, oleoresins, and essential oils that are used in poultry production (65–73) (Table 1). According to Madhupriya et al.' (68) explanation, PFAs are natural, less toxic, residue-free, and ideal feed additives for poultry compared with synthetic antibiotics.

**Table 1:** Phytogenic feed additives and ameliorative effects on broiler production.

**Feed additives**	**Level of supplementation**	**Findings**	**Sources**
Essential oils (Origanum genus)	300–600 g/kg	Increase in the average daily gain	(74)
Cinnamon	2 g/kg	Improve growth performance	(75)
Lippia Javanica leaf meal	5 g/kg	Improve daily gain and slaughter weight	(76)
The mixture of garlic and black pepper powder	5 g/kg) and 1 g/kg)	Increase in weight gain	(77)
Pennyroyal (Mentha pulegium L.)	2%	Increase in average daily gain	(78)
Neem (Azadirachta indica)	7 g/kg	Favorable influences on the immune	(79)
B. subtilis with enramycin	UBT-MO_2_/kg	Increase in body weight and relative weight of the thymus	(80)
Milk kefir	2%	Improvement on body mass and chicken consumption index	(81)

Herbs are flowering plants whose stem does not become woody and persistent, and are valued for their medical properties, flavor, and scent (51), whereas spices are pungent or aromatic substances of vegetable origin that are used as seasonings and preservatives (51). Botanicals or photobiotics are parts of a plant like roots, leaves, and barks, which are used to make drugs for medical use. Essential oils are any class of volatile oils obtained from plants; the possess the odor and other characteristic properties of plants and are used chiefly in the manufacture of perfumes, flavors, and pharmaceuticals (51). The most widely used herbs and spices for Phyto feed additives in poultry production are oregano, thyme, garlic, horseradish, chili, cayenne, pepper, peppermint, cinnamon, anise, clove, rosemary derivatives, citrus, and sage (68, 82).

A growing body of evidence has shown that supplementation of phytogenic feed additives in broilers' diet improve intestinal functions (83, 84), increase nitrogen retention and fiber digestibility, enhance growth performance (85), reduce inflammation (86), and improve anti-oxidative (51, 87) and antimicrobial activities (88) (Table 1). Altogether, the above findings suggest that PFAs have beneficial effects to improve performance and broiler health (54, 73, 75–77, 89, 90).

### Phytogenic Mode of Action

Studies have shown that the growth and health-promoting effects of PFAs are associated with their biological activities including antimicrobial, antioxidant, immunomodulatory, and anti-inflammatory (54, 68, 91–93). For instance, Superliv concentrate premix (SCP), AV/HGP/16 premix (AVHGP), and bacteriostatic herbal growth promoter (BHGP) have been increasing the feed efficiency of broilers by modulation of the muscle mTOR pathway and hepatic lipolytic programs; thus, they are promising for muscle protein synthesis and hepatic lipogenesis reduction (94). This is aligned with (95, 96) that have shown that PFAs modulate the expression of feeding-related hypothalamic neuropeptides and result in feed efficiency (FE) improvement. FE is also controlled by peripheral intermediary metabolism like lipid metabolism and protein synthesis-associated signaling pathways, which are modulated by bioactivities of PFAs.

PFAs also improve the palatability, digestibility, absorption of the feed nutrients, control animal intestinal microbiome structure, improve performance and feed quality through positively reflected of biological activities of plant secondary compounds with the action of antioxidative properties and slow microbial growth in poultry (97–99). In addition, they have been shown to enhance gut health by reducing bacterial colony populations, lessening fermentation products including ammonia and biogenic amines, decreasing the activity of the gut-associated lymphatic system, and increasing prececal nutrient digestion. Beneficial phytogenic compounds derived from their bioactive molecules are carvacrol, thymol, cineole, linalool, anethole, eugenol, capsaicin, allicin, allyl isothiocyanate, and piperine (65, 68). Most of these active secondary plant metabolites belong to the classes of isoprene derivatives, flavonoids, and glucosinolates, which act as antibiotics or antioxidants (100, 101).

### Organic Acids as Feed Additives

Organic acids are weak acids that have a carboxylic acid group (R-COOH) and nutritional values and antimicrobial effects in animal feeds (102–104). Organic acids have been used in animal feeds for many years because of the ban on the use of antibiotics (59). In line with these findings (15, 105, 106) reported that organic acids are considered as effective antibiotic alternatives in animal feeds. The most commonly used organic acids in the broilers' diet are acetic, butyric, citric, formic, propionic, malic, tartaric, and lactic acids (15, 28, 107).

The inclusion of organic acids in the broilers' diet has been shown to improve protein and carbohydrate digestibility (108), fight against pathogenic bacteria (105), and (106) enhance the feed conversion rate, nutrient utilization, and growth rate of broilers (109, 110).

### Organic Acid Mode of Action

Diets with poor protein quality have more indigestible proteins reaching the GIT, which end up with high protein fermentation (111). This high protein fermentation causes discomfort in the animal body and negatively affects its growth rate because of high volatile fatty acids and ammonia and production of other gases (112). Organic acids are good supplement alternatives in such types of feed to acidify the GIT environment (113) and improve nutrient utilization, which results in activeness of the protease enzyme. For example, Suiryanrayna and Ramana (114) reported stimulation of protein digestion by converting pepsinogen to pepsin by supplementation of organic acids. Moreover, organic acids reduce pH in the GIT, which enhances pepsin activity, and increases the digestibility of nitrogen, phosphorus, and other minerals (15, 115). These acid anions react with calcium, phosphorus, magnesium, and zinc, thus enhancing their digestibility. Peptides produced by pepsin proteolysis stimulate the release of gastrin and cholecystokinin hormones, which regulate protein digestion and absorption (116, 117).

Organic acids have been used as feed preservatives for protecting feed from microbial and fungal deterioration with the mechanism of acidification (118). These are a powerful tool in maintaining the health of the gastrointestinal tract of poultry, resulting in improvement in birds' production performance. For example, sanguinarine suppresses the growth of some harmful acid intolerance bacteria such as *E-coli, Salmonella spp*., and *Clostridium perfringens* that cause gastrointestinal distress (119), resulting in enhanced appetite and feed intake and improving growth (120). Reduction of competition for microbial nutrients in the host thereby increases the availability of nutrients (121), consequently increases BWG, and improves FCR (122, 123). Organic acids also affect the histological structure of the gastrointestinal tract; Consequently, improve nutrient absorption, maximized nutrient utilization efficiency, and improved growth performance (54). As a conclusion remark from different studies; organic acids and their salts are used to reduce a load of pathogenic microorganisms in the intestine, activate digestive enzymes, improve digestibility, and increase the absorption of nutrients, gut microflora function, and performance of chickens (Table 2).

**Table 2:** Organic acids, their derivatives, and ameliorative effects on broiler production.

**Organic acids**	**Level of supplementation**	**Finding**	**Sources**
CA	2%	Improve epithelial cell proliferation and villi height of gastrointestinal tract	(124)
CA, avilamycin	0.5 and 0.001%, respectively	Significantly increase growth performances at 35 days	(125)
BA	0.2%	Increase CW, breast meat yield, FCR, dressing % and reduce abdominal fat	(126, 127)
SB	0.6 and 1.2 g/kg	Increase ADG and FCR during 1–21 days period	(123)
N-butyric acid and 50% MB	250–7,000 mg/kg	Reduce *Salmonella Typhimurium* or *Clostridium perfringens*	(128)
MESB	800 mg/kg	Higher total body weight, daily gain and FCR at 35 days	(129)
PCB	0.3 g/kg	Increase weight gain	(130)
FA	5 g/kg	Increase BWG, dressing percentage and reduce FCR	(131)
KDF	5 g/kg	Increase BWG, dressing percentage and reduce FCR	(131)

*FCR, feed conversion ratio; CW, carcass weight; SB, sodium butyrate; MESB, microencapsulated sodium butyrate; ADG, average daily gain; PCB, protected calcium butyrate; CA, citric acid; BA, butyric acid; FA, formic acid,; KDF, potassium di-formate; BWG, body weight gain*.

### Prebiotics Feed Additives

Prebiotics are indigestible carbohydrates by the host animal but can be utilized by useful GIT microorganisms (54, 141–143). Prebiotics are found in different food sources such as oats, barley, dandelion greens, chicory, chia seeds, flax seeds, onion, garlic, almonds, and artichoke (144). Green algae (Chlorophyta) are also considered prebiotic because of the presence of water-soluble sulfated polysaccharides; the perform gut microbiota modulation and immunomodulation, and they have anti-oxidant, antibacterial, anti-hyperlipidemia, and anti-diabetic properties (145).

Potential prebiotics that have been fed to broilers include fructan, oligofructose, inulin, fructooligosaccharides, galactan, galactooligosaccharides, xylooligosaccharides (XOS), pectin, fiber components, and milk oligosaccharides (146–149). Refined functional carbohydrates (RFCs) including mannan-oligosaccharides (MOSs), β-glucan, and D-mannose, which are derived from the cell wall of *Saccharomyces cerevisiae*, are a readily available source of prebiotics for animal use (150). From these, mannan-oligosaccharides and fructooligosaccharides are the most common commercial feed nutrients in poultry feed production (151). In connection with their economic importance for producers, prebiotics also have no residual effect and do not develop any resistance for broiler product consumers (141).

Supplementation of prebiotics can improve growth performance and antibody titer against infectious bursal disease in broilers (Table 3) (133). Prebiotics are also useful for changing the microbial population of the intestine (31, 149, 152, 153); for example, dietary MOS (1g/kg) increase *Lactobacillus* and *Bifidobacterium* contents (154), increase the length of the villain (155), prevention of colon cancer, minimize disease-causing bacteria and increases daily weight gain (156, 157), and medical therapy of broiler (158). Generally, the beneficial effects of prebiotics are alteration of gut microorganisms that enable to increase their numbers, increase digestibility, reduce pathogenic bacteria, increase mineral and vitamin absorbability, maintain optimal intestinal pH, and maximize nutrients utilization (142, 143, 159).

**Table 3:** Prebiotics and their ameliorative effects on broiler production.

**Prebiotics**	**Level of supplementation**	**Finding**	**Sources**
FOS	0.25%	Improve productivity of broiler Increase lactobacillus in the ileum	(132)
MOS	0.05%	Improve productivity of broiler Increase lactobacillus in the ileum	(132)
MOS	1.5 g/kg	Improve WG and FCR Improve the antibody titer against IBD	(133)
IMO	5–10 g/ kg	Improve WG Increase feed conversion rate Increase the caecal populations of lactobacilli and bifidobacteria Decrease the caecal Escherichia coli Increase the caecal VFA	(134)
RFC	50–100 g/t	Improve ADG Decrease cecal Campylobacter counts The high dose also increases FBW	(135)
Autolyzed WY and YCW	1.5–2 g/kg	Improve BWG, FCR, and Meat yield Positive effect on ileal protein digestibility as well as trypsin and chymotrypsin activities	(136)

*ADG, average daily gain; FBW, final body weight; FOS, fructo-oligosaccharide; IMO, isomalto-oligosaccharide; IBD, infectious bursal disease; MOS, mannan-oligosaccharide; RFC, refined functional carbohydrate; VFA, volatile fatty acid; WY, whole yeast; YCW, yeast cell wall product*.

### Prebiotics Mode of Action

Prebiotics can affect host health in different ways, such as production of metabolites like lactic acid, microbial metabolism modification, and increase in epithelium cell integrity (160, 161). Prebiotics are used to modulate the ecosystem of gut elements including alteration of the intestinal microbiota, stimulation of the immune system, improvement of the epithelium, and regulation of interaction between the host and the intestinal microbiota (162).

Prebiotics could be a selective substrate for a limited number of beneficial bacteria to alter the colon microflora in favor of a healthier gastrointestinal environment (149, 152). For example, they serve as a substrate for endogenous beneficial bacteria, thus promoting competitive exclusion of pathogenic microbes and selective colonization by beneficial microbes (60). Mazanko et al. (159) also reported that a prebiotic feed supplement creates an unfavorable condition for pathogenic organisms by altering the pH of the intestine. It establishes a healthy microbial community in the intestine of broilers by enhancing the abundance of *Lactobacilli* and *Bifidobacteria* and reducing the titers of Coliform (163, 164). Bifidobacterium and Lactobacillus have manase enzymes; they selectively bind mannan oligosaccharides only for harmful bacteria, which normally do not have this enzyme (157). The effect of mannan oligosaccharides on broilers is increase in the daily weight gain of broilers by 4–8% (156, 157).

The sustainable ability of prebiotics in acidic environments and to remain resistant to distinct digestive enzymes in the small intestine make them an extraordinary tool to boost the growth of beneficial gut microbes that ferment them, leading to production of short-chain fatty acids, vitamins, and other fragmented molecules or some antibacterial substances such as bacteriocin against pathogenic microorganisms (165, 166). These fermented products of beneficial microbes due to prebiotic feed additives also improve the integrity of intestinal epithelial cells, which further increase the absorption of nutrients and enhance the growth performance of animals (115, 162).

**Figure 2:**
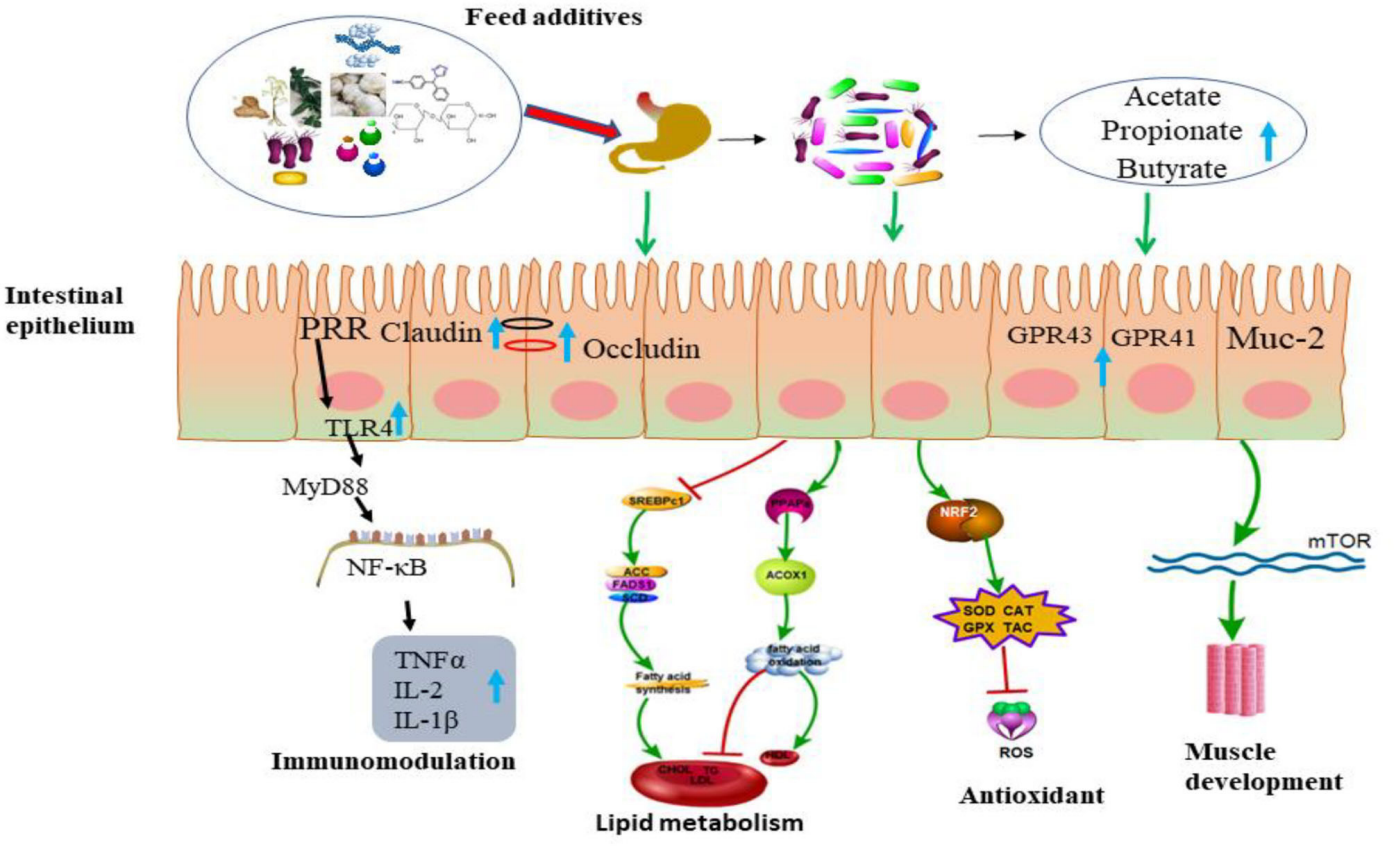
Schematic diagram of an alternative feed additive mode of action.

The modulation of the intestinal microbiota with prebiotic feed additives is associated with immune responses (162) (Figure 2). Oligosaccharides have been reported to present immunomodulatory beneficial effects on the gut, such as modifying clearance efficiency of pathogenic bacteria, activating T cell-dependent immune responses, and repression of pro-inflammatory cytokines (167, 168). Inhibiting pathogen colonization with prebiotics can decrease pathogen-associated molecular patterns, which are produced by pathogenic microorganisms (169). The produced molecule can be recognized by pattern recognition receptors (PRRs), including toll-like receptors (TLRs) and NOD-like receptors (NLRs), which are expressed on the surface of sentinel cells (170, 171). Once pattern recognition receptors (PRRs) recognize pathogen-associated molecular patterns (PAMPs), sentinel cells such as macrophages, epithelial cells, dendritic cells, and mast cells are activated and produce cytokines for regulation of further innate immune responses (171). They can be recognized by receptors of immune cells, consequently modulating host immunity systems.

### Probiotics Feed Additives

Probiotics are “live strains of strictly selected microorganisms that, when used in adequate amounts, confer a health benefit on the host” (172). Similarly, probiotics are beneficial bacteria that can fight pathogens in the gastrointestinal tract of chickens like subclinical necrotic enteritis (173), stimulate growth (174–176), and improve the immunity of the host (143, 177–179). Probiotics strains have been also providing feeding efficiency improvement, intestinal protection, antioxidant capacity and apoptosis (180), use of nutrients (181), energy digestibility, disappearance of non-starch polysaccharides (182), and microbial profile of cecum and litter (Table 4) (183).

**Table 4:** Probiotics and their ameliorative effects on broiler production.

**Probiotics**	**Level of supplementation**	**Finding**	**Sources**
Mixture of Bacillus licheniformis and Bacillus subtilis spores	0.05%	Significantly improve the FCR	(137)
Multi-strain probiotic (11 Lactobacillus strains)	1 g/kg	Increase FCR Improve BWG Increase the caecal populations of lactobacilli and bifidobacteria Increase the caecal VFA Decrease the caecal Escherichia coli	(134)
Protexin	2 g/kg	Improve the growth performance	(133)
Promax	1 g/L	Improve BW and the hemato-biochemical profile	(138)
Normosil	1 mL/kg	Increase the average daily gain Increase the level of blood erythrocytes Improve carcass quality	(139)
*Lact. lactis and L. plantarum*	10^9^ cfu/mL and 10^12^ cfu/mL, respectively	Lower the serum cholesterol, triglyceride, and total lipid contents Increase contents of blood glucose and total protein	(140)

*BWG, body weight gain; FCR, feed conversion rate; VFA, volatile fatty acid*.

Selection and use of microorganisms as feed additives are not an easy task; their risks, handling procedures, and adaptability to the environment should be considered. Some microbes will participate in the spread of antibiotic resistance (*enterococcus*) and produce toxin substances (*Bacillus cereus* strains) (184). The recommended dose for most probiotic strains is 10 × 9 colony-forming units of feed (CFU/KG). Care should also be taken when mixing probiotics. The water should be free from any disinfectant or chlorine. Administration or offering of a probiotic feed additive solution should be within 6–12 h after mixing with water. If animals are on antibiotic treatment, it is highly recommended that the treatment be withdrawn 24–48 h before administering probiotics (185).

The most used microorganisms as feed additives in poultry production are bacterial strains, mostly Gram-positive Bifidobacterium, and lactic acid bacteria groups such as *Bacillus, Enterococcus, Lactobacillus, Pediococcus, Streptococcus, Aspergillus, Candida, and Saccharomyces*. However, fungi and yeast strains are also used, mainly from the species *Saccharomyces cerevisiae* and *Kluyveromyces* (184, 186–188).

### Probiotics Mode of Action

The main modes of action of probiotics include antagonistic action toward pathogenic bacteria by secreting products that inhibit their development such as bacteriocins, organic acids, and hydrogen peroxide, and competitive exclusion by competing with bacteria for locations in the intestinal mucous membrane to adhere to nutrients (47). Lowering the gut pH through the volatile fatty acids and organic acids produced during probiotic product breakdown is the most common probiotic mode of action (189, 190). The low pH in the intestine suppresses the colonization of pathogens in the digestive tract, thereby competitively inhibiting the effects of pathogens (191). Probiotics are also used to modulate the intestinal microbiota, for immunomodulation, and to improve intestinal integrity (192, 193). Other principal mechanisms of probiotics are competition for binding sites where probiotics adhere to the intestinal epithelium wall, hindering competition and joining of pathogenic microorganisms; this higher concentration of the beneficial microbiota is also the driving force to have an advantage in the competition for nutrients (20). Findings showed that probiotics have nutritional effects, increasing fiber digestion and enzymatic activity in birds to be efficient in feed nutrient utilization (133). The finding of Wang et al. (194) stated that supplementation of broilers with *Bacillus subtilis* in the diet was more effective in performance in heat stress conditions through the immunity modulated by the microbiota.

### Enzymes as Feed Additives

Enzymes are catalysts of biochemical processes that are composed of proteins, amino acids with minerals, and vitamins (195). Enzymes are the most important and useful additives in the animal feed industry (196). They can be obtained from plants, animals, and microorganisms (197). Enzymes, as feed additives in broiler production, are produced by fermentation of fungi and bacteria and are used for maximization of feed conversion efficiency (FCE) (15). Although animals produce endogenous enzymes that are involved in digestion, they do not efficiently degrade feedstuff and take advantage of all their nutritional components; therefore, exogenous enzymes are supplemented to increase animal performance (195, 196, 198). Pectinases, amylases, cellulase, galactosidases, β glucanases, xylanases, associated enzyme phytases, proteases, and lipases are commonly used exogenous enzymes in the animal feed industry (Table 5) (196, 197, 207). These exogenous enzymes are mainly used in monogastric animals like poultry and swine (208).

**Table 5:** Enzymes, target substrates, and their benefits in broiler production.

**Broad classes of enzymes**	**Specific example**	**Substrate**	**Target feedstuff**	**Level of supplementation**	**Ameliorative Effect**	**Sources**
Carbohydrases	Xylanases	Arabinoxylans	Wheat, rye, triticale, barley, fibrous plant materials	3,200–24,000 IU/kg	Increase starch and nitrogen digestibility and improve AIDE	(199)
	α-Galactosidases	Oligosaccharides	Soybean meal, grain, legumes	50 mg/kg of diet	Improves intestinal histology and morphology	(200)
	α-amylase	Starch	Cereal grains, grain legumes	300–2,250 IU/kg	Improve the apparent ileal digestibility of energy	(201)
	β-Glucanases	β-Glucan	Barley, oats, and rye	20 IU/	Reduce viscosity, increases dry matter of digesta, and available energy	(202) (203) (204)
	β-Mannanase	Cell wall matrix (fiber components)	Plant-derived ingredients, fibrous plant materials	200–400 mg/kg		
	Cellulases			20 IU/kg		
	Hemicellulases			20 IU/kg		
	Pectinases			53 IU/ kg		
Proteases	Proteases	Proteins	All plant protein sources	30,000 IU/kg	Increase FI and FCR, increase N retention, reduce abdominal fat	(205)
Phytases	Phytates	Phytic acid	All plant-derived ingredients	500 – 1,500 FTU/kg	Increase FI, BW, FCR, CW, and GIT organs length	(206)

*AIDE, apparent ileal digestible energy; BW, body weight; CW, carcass weight; FCR, feed conversion ratio; FI, feed intake*.

The supplementation of enzymes for broilers has nutritionally, economically, and environmentally justifiable advantages (209). The use of enzymes in the chicken diet resulted in high feed utilization efficiency, reduction of digesta viscosity, enhanced digestion and absorption of nutrients, and increased feed intake and weight gain (18, 196, 210, 211). Xylanase has increased crude protein digestibility, feed intake, nitrogen and fiber absorption, and weight gain in broilers (211, 212). Phytases increase the utilization of phytate phosphorus in feeds (210). A multi-enzyme complex (Avizyme) composed of xylanases, proteases, and amylases is used to improve nutritional quality, reduce the viscosity of diets, increase body weight, decrease mortality, and increase the amount of net energy (213). It also improves the intestinal health of animals (214). Generally, different studies have reported that the use of exogenous feed enzymes in poultry diets is becoming familiar to overcome the adverse effects of anti-nutritional factors, and improve the digestion of dietary components and bird performance.

Enzymes must be active under physiological conditions prevailing in the animal's digestive tract and must complement the characteristics of dietary ingredients and additives to realize their functions (209, 215).

### Enzyme Mode of Action

Each enzyme has a different and interdependent mode of action; its use in combination with feed formulations must be carried out carefully to achieve maximum ameliorative effects (197). Broiler diets containing a large amount of NSP lead to increased digesta viscosity, thus depression in growth performance (216). Carbohydrase enzymes are added to broiler diets to overcome this type of difficulty, consequently improving nutrient utilization and increasing the productivity of birds. For example, hydrolysis of non-starch polysaccharides (NSPs) into smaller oligosaccharides with carbohydrase results in decrease in digesta viscosity and release of encapsulated nutrients (217). Produced small oligosaccharides during NSP hydrolysis could also have a prebiotic advantage (218). The hemicellulose in agro-industrial byproducts, particularly Palm kernel expeller (PKE), is partially hydrolyzed with enzyme treatment, thus obtaining oligosaccharides (DP <6) that have prebiotic-like effects. Based on Chen et al.'s (219) results, the untreated PKE contained 20.93 g/kg oligosaccharides, but after treatment, the oligosaccharide content increased to 28.91 and 59.71 g/kg for PKEENZ and SPKEENZ, respectively. Zhang et al. (220) also reported that smaller oligosaccharides such as xylooligosaccharide (XOS) come from hydrolysis of NSPs, which have been shown to have prebiotic-like effects.

Enzymes act on nutrients having main effects on substrates to which they are directed as well as having side effects. They initiate and control the rate of biological reactions by which substrates are changed into useful products (195). NSP hydrolysis products are fermented by beneficial bacteria such as Bifidobacter and Lactobacilli spp., thus, producing short-chain fatty acids (221). Increased SCFA concentration is often associated with increase in the population of beneficial bacteria and decrease in pathogenic bacteria (19). Some SCFAs are also used as an available energy source to the host for growth (222).

Supplementing glucose oxidase (GOD) in broilers has been reported to increase daily body weight gain, improve meat quality, and enhance digestive ability that is indicated by the nutrients' apparent digestibility and digestive enzymes (223). Different studies also confirm that the increase in body weight gain and FCR of broilers with commercial enzymes is due to the ileal digestibility of crude proteins (224), starch and fat (225), and improvement in ileal non-starch polysaccharide (NSP) digestibility (226). The content of secreted immunoglobulin A and transepithelial electrical resistance are also increased with the GOD supplement, which indicated an enhanced gut barrier. In the general context, dietary GOD supplement could improve the growth performance of broilers in two main mechanisms: 1) by enhancing the digestive function of the gut, which concluded from improved nutrients' apparent digestibility and digestive enzyme, and 2) by increasing the abundance of beneficial bacteria such as F. prausnitzii, Ruminococcaceae, and Firmicutes (223).

## Conclusion and Future Research Directions

The ban on certain antibiotics has promoted phytogenics, organic acids, prebiotics, probiotics, and enzymes as alternatives in broiler production. Antibiotic alternatives have comparable advantages to antibiotics to enhance the production performance and well-being of broilers without human health challenges. Moreover, using antibiotic alternatives can increase body weight, average daily gain, carcass weight, feed conversion ratio, and the nutritive value of feed ingredients, and enhance the gut health of broilers. The main provided effects of alternative feed additives includes immune-modulating, enhance digestion, improving nutrient availability, increase absorbability of nutrients, antimicrobial, antioxidant activity, enhancement of gut integrity, intestinal barrier function or improve intestinal health, nutrient for the host, and modulating the host gut microflora. These different modes of action suggest that there could be synbiotic, antagonistic, and synergistic or combative effects between alternatives or other feed nutrients. Therefore, use of alternative feed additives in broiler production should highly promoted and further investigations on interaction effects of combined additives, sub-additive, and with diet nutrient, efficiency of utilization, and level of inclusion could be mandatory.

## Author Contributions

HA carried out the organization and drafting of the manuscript. HZ and TW were involved more in technical editorial support of the drafted manuscript. All authors participated in the evaluation, editing, and approval of the final version of the manuscript.

## Funding

This study was funded by the Shandong Key Science and Technology Innovation Program (2019JZZY010704), the China Agriculture Research System-Beijing Team for Poultry Industry, and the Agricultural Science and Technology Innovation Program (ASTIP).

## Conflict of Interest

The authors declare that the research was conducted in the absence of any commercial or financial relationships that could be construed as a potential conflict of interest.

## Publisher's Note

All claims expressed in this article are solely those of the authors and do not necessarily represent those of their affiliated organizations, or those of the publisher, the editors and the reviewers. Any product that may be evaluated in this article, or claim that may be made by its manufacturer, is not guaranteed or endorsed by the publisher.
